# BMI as a Mediator of the Relationship between Muscular Fitness and Cardiometabolic Risk in Children: A Mediation Analysis

**DOI:** 10.1371/journal.pone.0116506

**Published:** 2015-01-15

**Authors:** Ana Díez-Fernández, Mairena Sánchez-López, Roberto Gulías-González, Blanca Notario-Pacheco, Jorge Cañete García-Prieto, Natalia Arias-Palencia, Vicente Martínez-Vizcaíno

**Affiliations:** 1 Social and Health Care Research Center, University of Castilla-La Mancha, Cuenca, Spain; 2 Faculty of Occupational Therapy, Speech Therapy and Nursing, University of Castilla-La Mancha, Talavera de la Reina, Toledo, Spain; 3 Faculty of Education, University of Castilla-La Mancha, Ciudad Real, Spain; 4 Facultad de Ciencias de la Salud, Universidad Autónoma de Chile, Talca, Chile; Children’s National Medical Center, Washington, UNITED STATES

## Abstract

**Objective:**

Muscular fitness levels have been associated with cardiometabolic risk in children, although whether body weight acts as a confounder or as an intermediate variable in this relationship remains controversial. The aim of this study was to examine whether the association between muscular fitness and cardiometabolic risk factors is mediated by body mass index (BMI).

**Design and Methods:**

Cross-sectional study using a sample of 1158 schoolchildren aged 8-11 years from the province of Cuenca, Spain. We measured anthropometrics and biochemical variables and we calculated a muscular fitness index as the sum of z-scores of handgrip dynamometry/weight and standing long jump, and we estimated a previously validated cardiometabolic risk index (CMRI). Linear regression models were fitted for mediation analysis to assess whether the association between muscular fitness and cardiometabolic risk was mediated by BMI.

**Results:**

Children with normal weight (NW) had a better cardiometabolic risk profile than their overweight (OW) or obese (OB) peers after controlling for muscular fitness. Marginal estimated mean±SE values for NW, OW and OB categories of CMRI were -0.75±0.06<0.84±0.10<2.18±0.16 in boys and -0.73±0.06<0.96±0.10<2.71±0.17 in girls, both p<0.001. Children with higher levels of muscular fitness had a better cardiometabolic risk profile (CMRI marginal estimated mean±SE 1.04±0.13>0.05±0.09>-1.16±0.13 for lower, middle and upper quartiles of muscular fitness in boys and 1.01±0.16>0.10±0.09>-1.02±0.15 in girls, both p<0.001), but differences disappeared when controlling for BMI. BMI acted as a full mediator between muscular fitness and most cardiometabolic risk factors (Sobel test z=-11.44 for boys; z=-11.83 for girls; p<0.001 in CMRI mediation model) and as a partial mediator in the case of waist circumference (Sobel test z=-14.86 for boys; z=-14.51 for girls; p<0.001).

**Conclusions:**

BMI mediates the association between muscular fitness and cardiometabolic risk in schoolchildren. Overall, good muscular fitness is associated with lower cardiometabolic risk, but particularly when accompanied by normal weight.

## Introduction

Although the definition of metabolic syndrome (MetS) in children remains controversial, it is generally accepted that MetS can be defined as a cluster of cardiometabolic disorders including insulin resistance or glucose intolerance, hypertension, dyslipidemia and central obesity [1], [2]. This clustering of risk factors tends to be consistent from childhood through adolescence and adulthood [3], [4], [5], and is considered a predictor of type 2 diabetes mellitus, cardiovascular disease and overall mortality [6], [7], [8].

Higher fitness levels are related to improved health in children and youth [9], [10]. The importance of muscular fitness (MF) is recognized in most current institutional recommendations for exercise helping to maintain and improve health status [10], [11] and preventing chronic diseases [12]. Several studies have shown an association between muscular strength and cardiometabolic risk in children and young adults [13], [14], [15] independent of cardiorespiratory fitness, adiposity and other confounding factors [16], [17].

In studies aimed to examine the relationship between MF and cardiometabolic risk, confounding or mediator variables have been usually controlled by multivariate methods such as ANCOVA [14], linear regression [15], or logistic regression [13], [16], depending on the objectives of the study and the characteristics of the dependent variable. Mediation analysis is a statistical procedure that can be used to clarify the processes underlying an association between two variables and the extent to which the association can be modified, mediated, or confounded by a third variable [18]. A mediation effect occurs when a third variable (the mediator) is responsible for the influence of a given independent variable on a given dependent variable.

The objective of this study was twofold: 1) to analyze the relationship between cardiometabolic risk factors and weight status and MF in schoolchildren and 2) to examine whether the association between MF and cardiometabolic risk factors was mediated by body mass index (BMI).

## Materials and Methods

### Study design and population

This was a cross-sectional analysis of baseline data from a cluster randomized trial (collected September-November 2010) aimed to assess the effectiveness of a physical activity program (MOVI-2) on preventing excess weight in schoolchildren [19]. The MOVI-2 study included 1158 schoolchildren aged 8 to 11 years from 20 public primary schools in the Province of Cuenca, Spain. The Clinical Research Ethics Committee of the Virgen de la Luz Hospital in Cuenca approved the study protocol. After obtaining the approval of the Director and Board of Governors (Consejo Escolar) of each school we sent a letter to the parents of all children in the 4th and 5th grades inviting them to a meeting. At this meeting the study objectives were outlined and written approval for their children’s participation was requested. Informative talks, in which the schoolchildren were asked to collaborate, were then held class by class.

### Measurement anthropometrics

Trained nurses collected anthropometric and blood pressure data. Data collection took place at the schools during September 2010. Weight and height were measured twice with a five-minute interval between measurements. Weight was measured to the nearest 100g using a calibrated digital scale (SECA Model 861; Vogel & Halke, Hamburg, Germany) with the children lightly dressed and without shoes. Height was measured to the nearest millimeter using a wall-mounted stadiometer, with the children standing straight against the wall without shoes, to align the spine with the stadiometer. The head was positioned so that the chin parallel to the floor. The mean of the two measurements of weight and height was used to calculate BMI as weight in kilograms divided by the square of the height in meters (kg/m^2^). Waist circumference (WC) was calculated as the average of two measurements taken with flexible tape at the natural waist (the midpoint between the last rib and the iliac crest). Fat mass percentage was estimated using a BC-418 bioimpedance analysis system (Tanita Corp., Tokyo, Japan) [20]. The mean of two readings taken in the morning under controlled temperature and humidity conditions, after urination and a 15-minute rest with the child being shoeless and fasting was used.

After these test and blood draw, diastolic and systolic blood pressure (DBP; SBP) were determined as the average of two measurements separated by a five-minute interval, with the child resting for at least five minutes before the first measurement. The child was seated in a quiet, calm environment, with the right arm in a semi-flexed position at heart level. Blood pressure was measured automatically using the OMRON M5-I monitor (Omron Healthcare Europe BV, Hoofddorp, Netherlands). Mean arterial pressure (MAP) was calculated using the following formula: DBP + (0.333 x (SBP-DBP)).

### Biochemical assessments

Blood samples were taken under standardized conditions between 8:15 and 9:00 a.m. after at least 12 hours fasting by puncturing the cubital vein. Before the extraction, fasting was confirmed by the child and his parents. When it was anticipated that the transfer of samples to the laboratory would take longer than 75 minutes they were centrifuged *in situ* and transferred refrigerated. Three aliquots of each sample were frozen, one for the determination of biochemical variables investigated in this study and the others for future analyses of which the parents were aware [19].

The following biochemical parameters were determined: triglycerides (TG), GPO-PAP (glycerolphosphate oxidase, peroxidase enzymatic method) and c-direct plus HDL.

Lipid profiles were determined over a 48-hour period using a MODULAR DPP system from Roche Diagnostics, and insulin levels were assessed using an Immulite 2000 double system platform of Siemens.

### Cardiometabolic risk assessment

We calculated a cardiometabolic risk index (CMRI) as the sum of the age-sex standardized scores of WC, TG-to-HDL-c ratio, MAP, and fasting insulin. The validity of this index has been previously tested using confirmatory factor analysis [21].

### Evaluation of fitness

MF was evaluated using handgrip and standing long jump (SLJ) tests. 1) Handgrip (maximum handgrip strength assessment) was assessed using the TKK 5401 Grip-D dynamometer (Takeya, Tokyo, Japan) (range: 5–100kg; precision: 0.1kg). The grip-span of the dynamometer was adjusted to the hand size of the schoolchildren. With the elbow in full extension the child had to press the dynamometer with the right hand for at least 2 seconds; the test was then repeated with the left hand. The test was performed twice and the maximum score for each hand was recorded in kilograms. The average of the maximum scores for both hands was used in analyses. 2) For the SLJ (lower body explosive strength assessment) the child jumped horizontally to achieve maximum distance, the best of three attempts was recorded in centimeters. These tests are validated and are included in the EUROFIT battery [22].

To avoid the potential biasing effect of body weight on the estimation of MF, handgrip was adjusted for body weight (in kg) in line with standard assumptions about morphologic effects as some authors have suggested [23]. In addition, a sex- and age-specific MF index (MF) was constructed from the sum of standardized z-scores on the handgrip/weight and SLJ tests.

Cardiorespiratory fitness (CRF) was assessed by the 20-m shuttle run test. Children were required to run between two lines 20m apart, keeping pace with audio signals from a pre-recorded CD. The initial speed was 8.5km/h and this was increased by 0.5km/h every minute (1 minute equals one stage). The children were encouraged to keep running as long as possible throughout the test and it ended when the child failed to reach the line in time with the audio signal on two consecutive occasions. We recorded the last half-stage completed as an indicator of CRF [24].

### Pubertal development

Sexual maturation was assessed with a standardized procedure in which parents identify the pubertal status of their child using pictures. Pubertal status was classified according to the five stages of pubertal maturity defined by Tanner and Whitehouse [25].

### Statistical analysis

The distribution of continuous variables was checked for normality before further analysis; fasting insulin and TG/HDL-c data were normalized with a natural logarithm transformation. Partial correlation coefficients were estimated to examine the relationship between cardiometabolic risk factors and BMI, MF, dynamometry/weight and SLJ, controlling for age.

MF was categorized as lower Q (first quartile), middle Q (second and third quartiles) or upper Q (fourth quartile). Children were classified as normal weight, overweight or obese according to gender- and age-specific BMI cut-offs [26]. ANCOVA models were used to assess differences in the cardiometabolic risk factors across BMI and MF categories, controlling for age (model 1); and with further adjustment for MF or BMI depending on the fixed factor (model 2), by sex. Pairwise *post-hoc* hypotheses were tested using the Bonferroni correction for multiple comparisons.

The same analyses were used to assess differences in the cardiometabolic risk factors and CMRI levels across categories of dynamometry/weight and SLJ, controlling for age (model 1); and with further adjustment for dynamometry/weight, SLJ or BMI depending on the fixed factor (model 2), by sex; the results are shown in S1 File.

As there is no consensus when presenting data from muscular fitness tests and the consideration of body size, where several approaches have been used [23], we also performed the analysis of the entire sample adjusting SLJ and dynamometry by an allometric normalization method proposed by Jaric [27] showing these results in S1 File and also regressing body mass on the fitness phenotypes.

Linear regressions models were fitted according to the procedures outlined by Baron and Kenny [28] to assess whether the association between MF and cardiometabolic risk factors and CMRI was mediated by BMI. The first equation regressed the mediator (BMI) on the independent variable (MF). The second equation regressed the dependent variable (CMRI, WC, log TG/HDL-c ratio, MAP or log fasting insulin) on the independent variable.

The third equation regressed the dependent variable on both the independent variable and the mediator variable. Because of the close relationship between WC and BMI, WC was removed from CMRI when the mediation analysis included both variables.

The following criteria for mediation were used: 1) the independent variable is significantly related to the mediator; 2) the independent variable is significantly related to the dependent variable; 3) the mediator is significantly related to the dependent variable; and 4) the association between the independent and dependent variables is attenuated when the mediator is included in the regression model. We also assessed mediation using the steps outlined by Sobel [29]: first we estimated the attenuation or indirect effect (i.e. the effect of the independent variable on the mediator from the first regression model multiplied by the effect of the mediator on the dependent variable obtained from the third regression model) and then we divided the indirect effect by its standard error and performed a Z test of the null hypothesis that the indirect effect is equal to zero.

Because of the strong association between MF and CRF [15], [30], the mediation analysis was repeated controlling for CRF in all cardiometabolic risk factors.

Mediation analyses models were repeated with dynamometry/weight and SLJ as independent variables (results are shown in S1 File).

A bilateral criterion for statistical significance of *P* ≤ 0.05 was used. All statistical analyses were performed using the software IBM SPSS 20 for Macintosh (SPSS, Inc., Chicago, IL).

## Results

We invited 1596 schoolchildren to participate in the study and 1158 (72.6%) accepted; there were no age or sex differences between children who agreed to participate and those who did not. Table 1 summarizes participants’ characteristics by sex. We found no sex differences in CMRI, BMI or MF.

**Table 1:** Characteristics of the study sample.

	**Total (n = 1158)**	**Boys (n = 587)**	**Girls (n = 571)**	**p-value**
Age (yr)	9.5(0.71)	9.5(0.73)	9.5(0.69)	0.48
Tanner Stage	1.6(0.65)	1.5(0.59)	1.7(0.70)	**<0.001**
Height (cm)	139.6(6.99)	139.5(6.90)	139.6(7.09)	0.82
Weight (kg)	37.4(9.22)	37.7(9.61)	37.0(8.79)	0.23
BMI (kg/m^2^)	19.0(3.68)	19.2(3.81)	18.8(3.54)	0.14
Fat mass (%)	25.4(6.79)	23.9(7.14)	26.8(6.09)	**<0.001**
Dynamometry (kg)	14.6(3.37)	15.4(3.45)	13.8(3.08)	**<0.001**
Dynamometry/weight (kg)	-0.40(0.09)	-0.38(0.09)	-0.42(0.09)	**<0.001**
Standing Long Jump (cm)	114.5(19.67)	119.6(19.57)	109.3(18.37)	**<0.001**
Muscular fitness^a^	-0.0013(1.70)	0.0011(1.72)	-0.0036(1.68)	0.96
Cardiorespiratory fitness^b^	3.5(1.69)	4.1(1.83)	2.9(1.25)	**<0.001**
SBP (mmHg)	101.2(9.33)	102.7(9.38)	99.6(9.03)	**<0.001**
DBP (mmHg)	62.4(7.21)	62.4(7.30)	62.3(7.13)	0.85
MAP (mmHg)	75.3(7.23)	75.8(7.31)	74.7(7.10)	**0.012**
Fasting glucose (mg/dl)	83.57(7.03)	84.66(7.66)	82.46(6.13)	**<0.001**
Fasting insulin (mg/dl)	0.83(0.23)	0.80(0.23)	0.87(0.23)	**<0.001**
Waist circumference (cm)	67.6(9.38)	68.2(9.73)	67.0(8.98)	**0.030**
Triglycerides (mg/dl)	66.26(34.43)	63.00(33.51)	69.58(35.06)	**<0.001**
HDL-c (mg/dl)	60.49(13.53)	61.95(13.97)	59.00(12.90)	**<0.001**
TG/HDL-c (mg/dl)	0.026(0.25)	-0.009(0.25)	0.061(0.24)	**<0.001**
CMRI	-0.006(1.70)	-0.007(1.70)	-0.004(1.70)	0.98

Data are presented by mean(SD). Abbreviations: BMI = body mass index; MAP = mean arterial pressure (DBP + {0.333 x (SBP—DBP)}); Log fasting insulin = logarithm of fasting insulin; Log TG/HDL-c = logarithm of triglyceride to high-density lipoprotein cholesterol ratio; CMRI = cardiometabolic risk index (sum of standardized z score of its components: log fasting insulin, waist circumference, mean arterial blood pressure and log TG/HDL-c).

^a^ Sum of the standardized z score of dynamometry/weight and standing long jump

^b^ Measured by 20-m shuttle run test (stage). p-value less than or equal to 0.05.

Partial correlations between BMI, MF, dynamometry/weight, SLJ and cardiometabolic risk factors and CMRI controlling for age are shown in Table 2. BMI was positively associated with all the cardiometabolic risk factors investigated and MF, dynamometry/weight and SLJ were negatively associated with the same set of cardiometabolic risk factors (*p*<0.05). Similar results were obtained when we included Tanner stage as a covariate, but in these models there were no significant relationships between MAP and MF, dynamometry/weight or SLJ (data not shown).

**Table 2:** Partial correlations coefficients (*r*) between body mass index, muscular fitness, dynamometry/weight and standing long jump with cardiometabolic risk factors controlling for age.

		**MAP**	**Log insulin**	**Log TG/HDL-c**	**WC**	**CMRI**
BMI	Total	0.35**	0.53**	0.46**	0.94**	0.79**
	Boys	0.36**	0.53**	0.48**	0.94**	0.79**
	Girls	0.36**	0.56**	0.46**	0.93**	0.80**
MF^a^	Total	-0.16**	-0.30**	-0.28**	-0.56**	-0.46**
	Boys	-0.19**	-0.36**	-0.33**	-0.59**	-0.51**
	Girls	-0.13*	-0.25**	-0.23**	-0.52**	-0.41**
Dynamometry /weight	Total	-0.15**	-0.35**	-0.32**	-0.58**	-0.48**
	Boys	-0.20**	-0.36**	-0.32**	-0.62**	-0.53**
	Girls	-0.13*	-0.30**	-0.28**	-0.60**	-0.45**
SLJ	Total	-0.10*	-0.24**	-0.23**	-0.32**	-0.29**
	Boys	-0.14**	-0.27**	-0.25**	-0.40**	-0.37**
	Girls	-0.09*	-0.14*	-0.14*	-0.29**	-0.22**

Abbreviations: BMI = body mass index; MF index = muscular fitness; MAP = mean arterial pressure (DBP + {0.333 x (SBP—DBP)}); Log insulin = logarithm of fasting insulin; Log TG/HDL-c = logarithm of triglyceride to high-density lipoprotein cholesterol ratio; WC = waist circumference; CMRI = cardiometabolic risk index (sum of standardized z score of its components: log insulin, waist circumference, MAP and log TG/HDL-c); SLJ = standing long jump.

^a^Muscular fitness: measured by the sum of standardized z score of dynamometry/weight and standing long jump.

p-value = **p<0.001 and *p<0.05.

Mean differences in CMRI and cardiometabolic risk factors by BMI and MF categories, controlling for age (model 1) are shown in Table 3 (boys) and Table 4 (girls). Cardiometabolic risk factors were significantly worse in children with excess weight and significantly better in children with higher MF. Most post-hoc pairwise comparisons of means indicated statistically significant differences in boys and girls (normal weight < overweight < obesity for BMI categories and lower Q > middle Q > upper Q for MF). Similar results were obtained when MF was included in the ANCOVA models as a covariate with BMI categories as the fixed factor (model 2), but when BMI was added to the ANCOVA models as covariate with MF categories as the fixed factor the effect of MF disappeared.

**Table 3:** Mean differences in cardiometabolic risk factors by body mass index and muscular fitness categories in boys.

	**Body Mass Index**								**Muscular fitness**							
	**Model 1**				**Model 2**				**Model 1**				**Model 2**			
	***NW***	***OW***	***OB***	***p-value***	***NW***	***OW***	***OB***	***p-value***	***Lower Q***	***Middle Q***	***Upper Q***	***p-value***	***Lower Q***	***Middle Q***	***Upper Q***	***p-value***
n =	353	152	60		353	152	60		133	253	143		133	253	143	
MAP (mmHg)	74.1 ±0.35	77.4 ±0.54	82.0 ±0.87	**<0.001**	74.2 ±0.37	77.3 ±0.56	81.6 ±0.94	**<0.001**	77.3 ±0.60^M^	76.0 ±0.42	73.9 ±0.60	**<0.001**	75.3 ±0.61	76.0 ±0.40	76.0 ±0.61	0.576
Log Insulin (mg/dl)	0.72 ±0.01	0.88 ±0.01	1.04 ±0.02	**<0.001**	0.73 ±0.01	0.86 ±0.01	0.99 ±0.02	**<0.001**	0.90 ±0.02	0.80 ±0.01	0.70 ±0.01	**<0.001**	0.80 ±0.01	0.80 ±0.01	0.78 ±0.01	0.700
Waist circum- ference (cm)	62.6 ±0.27	74.5 ±0.42	86.0 ±0.67	**<0.001**	63.3 ±0.27	73.6 ±0.41	83.8 ±0.69	**<0.001**	75.3 ±0.68	68.2 ±0.48	61.1 ±0.68	**<0.001**	68.5 ±0.29	68.3 ±0.18	67.8 ±0.29	0.185
Log TG/HDL-c (mg/dl)	-0.09 ±0.01	0.07 ±0.01	0.26 ±0.02	**<0.001**	-0.08 ±0.01	0.06 ±0.01	0.23 ±0.03	**<0.001**	0.08 ±0.02	0.002 ±0.01	-0.12 ±0.02	**<0.001**	-0.01 ±0.02	0.003 ±0.01	-0.03 ±0.02	0.270
CMRI	-0.87 ±0.06	0.99 ±0.09	2.56 ±0.15	**<0.001**	-0.75 ±0.06	0.84 ±0.10	2.18 ±0.16	**<0.001**	1.04 ±0.13	0.05 ±0.09	-1.16 ±0.13	**<0.001**	0.01 ±0.10	0.06 ±0.06	-0.18 ±0.10	0.090

Data are presented as marginal estimated mean ± SE.

MAP = mean arterial blood pressure (DBP + {0.333 x (SBP—DBP)}); Log Insulin = logarithm of fasting insulin; Log TG/HDL-c = logarithm of triglyceride to high density lipoprotein cholesterol ratio. CMRI = cardiometabolic risk index.

Categories of body mass index (BMI) are Normal Weight (NW), Overweight (OW) and Obesity (OB) according to gender-and-age-specific cut-offs defined by Cole and Lobstein. Categories of muscular fitness (an index was measured by the sum of the standardized z score of dynamometry/weight and standing long jump) are Lower Q (representing 1^st^ quartile), Middle Q (2^nd^ and 3^rd^ quartiles), and Upper Q (4th quartile).

Model 1 controlling for age. All the pairwise mean comparisons using Bonferroni post-hoc test were statistically significant (p<0.001) (NW<OW<OB for BMI categories; and Lower Q>Middle Q>Upper Q for muscular fitness), except for superscript letter.

Model 2 further adjustments for muscular fitness to BMI (NW<OW<OB); and for BMI to muscular fitness. All the pairwise mean comparison using Bonferroni post-hoc test were statistically significant (NW<OW<OB for BMI categories).

**Table 4:** Mean differences in cardiometabolic risk factors by body mass index and muscular fitness categories in girls.

	**Body Mass Index**								**Muscular fitness**							
	**Model 1**				**Model 2**				**Model 1**				**Model 2**			
	***NW***	***OW***	***OB***	***p-value***	***NW***	***OW***	***OB***	***p-value***	***Lower Q***	***Middle Q***	***Upper Q***	***p-value***	***Lower Q***	***Middle Q***	***Upper Q***	***p-value***
n =	342	147	57		342	147	57		93	324	113		93	324	113	
MAP (mmHg)	73.1 ±0.34	76.7 ±0.56	81.1 ±0.88	**<0.001**	73.0 ±0.35	76.9 ±0.59	81.7 ±0.94	**<0.001**	76.8 ±0.59	74.3 ±0.41^G^	73.7 ±0.59	**<0.001**	74.5 ±0.62	74.7 ±0.39	75.4 ±0.59	0.546
Log Insulin (mg/dl)	0.80 ±0.01	0.96 ±0.01	1.17 ±0.02	**<0.001**	0.80 ±0.01	0.96 ±0.01	1.16 ±0.03	**<0.001**	0.98 ±0.02^G^	0.85 ±0.01	0.81 ±0.02	**<0.001**	0.86 ±0.01	0.87 ±0.01	0.89 ±0.01	0.351
Waist circum- ference (cm)	62.2 ±0.27	73.6 ±0.44	83.7 ±0.70	**<0.001**	62.4 ±0.27	72.7 ±0.44	82.2 ±0.71	**<0.001**	74.5 ±0.64	65.9 ±0.45	61.6 ±0.65	**<0.001**	67.6 ±0.31^M^	66.9 ±0.20^G^	66.4 ±0.30	**0.038**
Log TG/HDL-c (mg/dl)	0.003 ±0.01	0.11 ±0.02	0.34 ±0.03	**<0.001**	0.007 ±0.01	0.10 ±0.02	0.34 ±0.03	**<0.001**	0.16 ±0.02	0.03 ±0.01^G^	0.01 ±0.02	**<0.001**	0.07 ±0.02	0.05 ±0.01	0.08 ±0.02	0.442
CMRI	-0.79 ±0.06	1.03 ±0.10	2.83 ±0.16	**<0.001**	-0.73 ±0.06	0.96 ±0.10	2.71 ±0.17	**<0.001**	1.01 ±0.16	0.10 ±0.09	-1.02 ±0.15	**<0.001**	0.07 ±0.02	0.05 ±0.01	0.08 ±0.02	0.442

Data are presented as marginal estimated mean ± SE.

MAP = mean arterial blood pressure (DBP + {0.333 x (SBP—DBP)}); Log Insulin = logarithm of fasting insulin; Log TG/HDL-c = logarithm of triglyceride to high density lipoprotein cholesterol ratio. CMRI = cardiometabolic risk index.

Categories of body mass index (BMI) are Normal Weight (NW), Overweight (OW) and Obesity (OB) according to gender-and-age-specific cut-offs defined by Cole and Lobstein. Categories of muscular fitness (an index was measured by the sum of the standardized z score of dynamometry/weight and standing long jump) are Lower Q (representing 1^st^ quartile), Middle Q (2^nd^ and 3^rd^ quartiles), and Upper Q (4th quartile).

Model 1 controlling for age. All the pairwise mean comparisons using Bonferroni post-hoc test were statistically significant (p<0.001) (NW<OW<OB for BMI categories; and Lower Q>Middle Q>Upper Q for muscular fitness), except for superscript letters.

Model 2 further adjustments for muscular fitness to BMI (NW<OW<OB); and for BMI to muscular fitness. All the pairwise mean comparison using Bonferroni post-hoc test were statistically significant (NW<OW<OB for BMI categories), except for superscript letters in muscular fitness categories.

Analysis of differences in CMRI and cardiometabolic risk factors among SLJ and dynamometry/weight categories showed that the children with better MF had lower cardiometabolic risk (controlling for age); but when BMI was added as a covariate (model 2) the differences disappeared in both sexes (A–D Tables in S1 File). Similar results have been found when SLJ, dynamometry and MF index were normalized by allometric parameters (E–G Tables in S1 File) and also when we used residuals of body mass regression on the fitness phenotypes (data not shown).

### Mediation analysis

**Figure 1:**
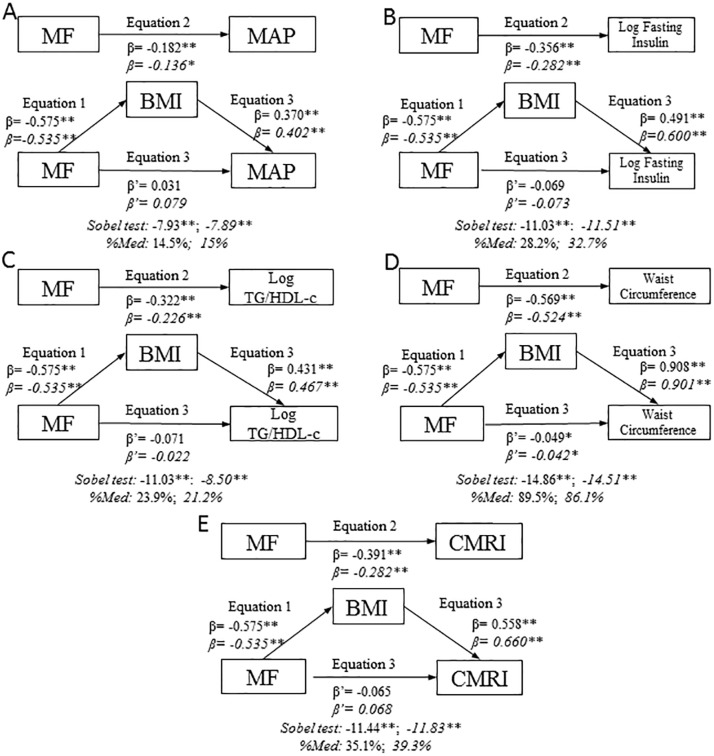
BMI mediation models of the relationship between muscular fitness and cardiometabolic risk variables, controlling for age, by sex. A: MAP; B: log fasting insulin; C: log TG/HDL-c; D: WC; E: CMRI. Data in roman type refer to boys. %Med: Percentage mediated by proposed mediator. Data in italics refer to girls. **p≤0.001; *p≤0.05.

We tested BMI as a potential mediator of the relationship between MF and MAP (Fig. 1A). In the first regression equation MF was negatively associated with BMI (*p≤*0.001). In the second equation MF was also negatively associated with MAP (*p≤*0.001). Finally, in the third equation, with MF and BMI both included in the model BMI was positively associated with MAP (*p≤*0.001) and MF was positively associated with CMRI in boys and girls although the associations were not statistically significant. These results suggest that the effect of MF on MAP was fully mediated by BMI. Using the Sobel test for mediation it was estimated that in boys 14.5% (z=-7.93; *p≤*0.001) and in girls 15% (z=-7.89; *p≤*0.001) of the total effect of MF on MAP was mediated by BMI.

Analysis of BMI as a potential mediator of the relationship between MF and log fasting insulin (Fig. 1B), log TG/HDL-c (Fig. 1C) and CMRI (Fig. 1E) produced similar results so BMI may be considered a full mediator of the effect of MF on these biometric parameters and index in both sexes. It was estimated that in boys 28.2% (z=-11.03; *p≤*0.001) of the effect of MF on log fasting insulin was mediated by BMI; 23.9% (z=-11.03; *p*≤0.001) of the effect of MF on log TG/HDL-c was mediated by BMI and 35.1% (z=-11.44 *mp*≤0.001) of the effect of MF on CMRI was mediated by BMI. In girls the percentage of total effect of MF mediated by BMI were 32.7% (z=-11.51; *p*≤0.001) for log fasting insulin, 21.1% (z=-8.50; *p≤*0.001) for log TG/HDL-c, and 39.3% (z=-7.89; *p≤*0.001) for CMRI.

BMI partially mediated the association between MF and WC (Fig. 1D). In boys 89.5% (z = 14.86; *p≤*0.001) of the effect of MF on WC was mediated by BMI; the corresponding percentage in girls was 86.1% (z=-14.51; p≤0.001).

When the above mediation models were estimated whilst controlling for CRF the results remain very similar, showing BMI as a full mediator of the association between MF and cardiometabolic risk factors in both boys and girls, with the exception that BMI only partially mediated the relationship between log fasting insulin and CMRI in girls (data not shown).

We also tested the analysis with the entire sample using sex and age as adjustment factors, and results did not differ (data not shown).

BMI was also a complete mediator of the relationship between SLJ and all cardiometabolic risk factors, except for CMRI and WC in boys; BMI was a partial mediator of the association between these factors and SLJ. Similarly BMI was a complete mediator of the relationship between dynamometry/weight and all cardiometabolic risk factors, except for WC in boys, and CMRI, WC and MAP in girls (A and B Figs. in S1 File).

## Discussion

The present study is the first to investigate the involvement of body composition in the relationship between MF and cardiometabolic risk in schoolchildren using mediation analysis. Children with normal weight had a better cardiometabolic risk profile than their overweight and obese peers after adjustment for age and MF; conversely higher levels of MF were associated with lower cardiometabolic risk independent from age, but after adjustment for BMI this association disappeared. Taken together these results suggest that BMI acts as a full mediator of the relationship between MF and cardiometabolic risk in boys and girls.

### Obesity and cardiometabolic risk

A recent systematic review and meta-analysis describing the association between BMI, sex and cardiometabolic risk factors reported that overweight and obese children have higher blood pressure and higher levels of triglycerides, total cholesterol, low density lipoprotein (LDL) cholesterol and fasting insulin [31]. Our data, like those from studies that have used similar analytical techniques [32], [33], [34], support the conclusions of this review. Overall all cardiometabolic parameters and CMRI increased with BMI even after controlling for MF, y their statistical significance remains virtually unchanged after this adjustment. These findings, along with those a similar previous paper that also evidenced the mediator role of BMI in the association of aerobic capacity with cardiometabolic risk [35] reinforce the importance of setting the reduction of excess of weight as a goal of physical activity programs, particularly in overweight and obese people.

### MF and cardiometabolic risk

Muscle strength has been associated with cardiometabolic risk factors in adults [23], and even with overall mortality [30]. In children and adolescents higher MF has been negatively associated with cardiovascular disease and metabolic risk factors in several studies [13], [14], [16], [17], [36], [37]. Our data partially support these findings, because although higher MF was associated with lower cardiometabolic risk in boys and girls, this inverse association disappeared when the effects of BMI were controlled. Benson *et al*. [38] reported that children with moderate or high upper body muscular strength had 98% less chance of developing insulin resistance than children with low strength after adjusting for maturation, WC and BMI. Magnussen *et al*. reported that in children and adolescents muscular power and endurance were inversely associated with clustered cardiovascular disease risk score after controlling for BMI, although the association was not significant for muscular strength [15]. There were some methodological differences between these studies that may partially explain the difference in results. Benson *et al*. had a smaller study sample (n = 126) and upper body muscular strength was measured as 1RM for bench press whereas Magnussen *et al*. indexed muscular endurance as push-ups performed in a 30-second period and in our study MF was calculated as sum of the standardized z-scores of handgrip/weight test and SLJ test; although when we used handgrip or SLJ as indicators of muscular strength separately the results were similar.

There is a lack of consensus regarding the more adequate methodology for normalizing indices of muscle strength for differences in human body. Most of the published data are body size dependent, thus comparisons between subjects are precluded. It has been suggested that allometric rather than ratio scaling are more suitable methods for normalization of muscular fitness for body size [27]. In addition to adjustment for body weight, two methods to correct the effect of allometry have been used in this study: the use of an allometric parameter and the use of residuals of regressing body size on muscular fitness test. Our data support that for studies not in laboratory settings but in population-based samples the election of the approach for the normalizing indices of muscular strength is not a relevant issue probably because of, as has been pointed out [39], allometric parameters depends on the type of the physical performance test.

### BMI as a mediator between MF and cardiometabolic risk factors

A strong evidence for an inverse association with muscular fitness has been recently described for adiposity and cardiometabolic risk in a meta-analysis [37]. Overall our mediation analysis revealed that in both sexes the effect of MF on cardiometabolic risk was fully mediated by BMI, with the only exception of waist circumference, in which BMI acted as a partial mediator.

Although some studies have shown that children and adolescents with excess weight and high MF have lower metabolic risk than their peers with excess weight and low MF [13], [14], our mediation analysis leads us to question whether good MF counteracts the negative cardiometabolic consequences of excessive body weight in children.

As we commented above, an earlier study by our group provided evidence that BMI mediates the influence of cardiorespiratory fitness on cardiometabolic risk [35]; the results of this study provide further evidence that BMI is an intermediate variable that should always be considered when analyzing the relationship between physical fitness and cardiometabolic risk. This means that you can expect a weak impact on cardiometabolic health of those physical activity programs that improving fitness, fail on diminish the adiposity. But this also means that you can expect reductions of weight associated to both cardiorespiratory and muscular fitness. Finally, this not means that programs for reduction of excess of weight based in strategies other than improvement of physical fitness will achieve improvements in cardiometabolic risk.

Clinical guidelines should take into account the results of this study and to recommend the prescription of physical activity for children with excess of weight, particularly when they are at higher cardiometabolic risk levels. This is probably the best way of preventing cardiovascular disease and diabetes in the coming years.

## Limitations

The primary limitation on our study was the cross-sectional design, which prevented us from making causal inferences; data obtained from prospective studies might provide confirmation of our findings. The generalizability of our results may be limited because of the complexity of influences on children’s cardiometabolic profile (regional variability of MetS prevalence, genetics and environmental influences etc.). However the sample size in this population-based study and the apparent representativeness of the sample are reasons to be confident of the validity of our results. Changes in levels of sex hormones due to maturation may affect muscle mass and consequently muscular strength but our sample was fairly homogeneous with respect to sexual maturation, most of the children in our study were in the first and second Tanner stages (86.8% of the sample; n = 249) so this probably did not affect our results.

We are aware that SLJ and maximum handgrip strength tests are not the best estimators of muscular fitness but both have demonstrated to be a cost-effective way of estimate muscular fitness [14] in population based studies in youth [40].

Although BMI has evidenced to be a mediator variable in the relationship between MS and cardiometabolic risk factors, the determination coefficient of the regression models suggest that other factors (i.e. amount of physical activity) could be influencing this relationship; future studies testing multiple moderator/mediator models by using structural equations or other sophisticated regression approaches will be useful to clarify the potential mediator, moderator or confounder role of these factors.

## Conclusions

Our findings have important implications clinical and public health point of view because they indicate that the physical activity programs aimed at improving muscular fitness in children only result in improvements in cardiometabolic risk if they were associated to reductions in adiposity, particularly in obese children. As a consequence, we should include weight reduction as an additional goal of resistance programs and not only improvements in muscular fitness.

## Supporting Information

S1 File Contains the following files: **A Fig.** BMI mediation models of the relationship between dynamometry/weight and cardiometabolic risk factors, controlling for age, by sex. **B Fig.** BMI mediation models of the relationship between standing long jump and cardiometabolic risk factors, controlling for age, by sex. **A Table.** ANCOVA model testing mean differences in cardiometabolic risk factors by body composition and dynamometry/weight categories in boys. **B Table.** ANCOVA model testing mean differences in cardiometabolic risk factors by body composition and dynamometry/weight categories in girls. **C Table.** ANCOVA model testing mean differences in cardiometabolic risk factors by body composition and standing long jump categories in boys. **D Table.** ANCOVA models testing mean differences in cardiometabolic risk factors by body composition and standing long jump categories in girls. **E Table.** ANCOVA model testing mean differences in cardiometabolic risk factors by body mass index and adjusted standing long jump categories. SLJ was adjusted by allometric parameters defined by Jaric (SLJ/weight^0^). **F Table.** ANCOVA model testing mean differences in cardiometabolic risk factors by body mass index and adjusted dynamometry categories. Dynamometry was adjusted by allometric parameters defined by Jaric (dynamometry/weight^0.67^). **G Table.** ANCOVA model testing mean differences in cardiometabolic risk factors by body mass index and adjusted muscular fitness categories. Muscular fitness = sum of standardized z score of dynamometry/weight^0.67^ and SLJ/weight^0^, according to allometric parameters defined by Jaric.
(ZIP)
