# Supplementary material for: BMI as a Mediator of the Relationship between Muscular Fitness and Cardiometabolic Risk in Children: A Mediation Analysis
Source: PLoS One. 2015 Jan 15;10(1):e0116506. doi: 10.1371/journal.pone.0116506 (PMC4295865; doi:10.1371/journal.pone.0116506)
Supplement: S1 File — B Fig. BMI mediation models of the relationship between standing long jump and cardiometabolic risk factors, controlling for age, by sex. A Table. ANCOVA model testing mean differences in cardiometabolic risk factors by body composition and dynamometry/weight categories in boys. B Table. ANCOVA model testing mean differences in cardiometabolic risk factors by body composition and dynamometry/weight categories in girls. C Table. ANCOVA model testing mean differences in cardiometabolic risk factors by body composition and standing long jump categories in boys. D Table. ANCOVA models testing mean differences in cardiometabolic risk factors by body composition and standing long jump categories in girls. E Table. ANCOVA model testing mean differences in cardiometabolic risk factors by body mass index and adjusted standing long jump categories. SLJ was adjusted by allometric parameters defined by Jaric (SLJ/weight0). F Table. ANCOVA model testing mean differences in cardiometabolic risk factors by body mass index and adjusted dynamometry categories. Dynamometry was adjusted by allometric parameters defined by Jaric (dynamometry/weight0.67). G Table. ANCOVA model testing mean differences in cardiometabolic risk factors by body mass index and adjusted muscular fitness categories. Muscular fitness = sum of standardized z score of dynamometry/weight0.67 and SLJ/weight0, according to allometric parameters defined by Jaric. (ZIP) [file pone.0116506.s001.zip › S1 File/Tables A-G.docx]

**Table A. ANCOVA model testing mean differences in cardiometabolic risk factors by body composition and dynamometry/weight categories in boys.**

|  | **Body Mass Index** | | | | | | | | **Dynamometry/weight** | | | | | | | |
| --- | --- | --- | --- | --- | --- | --- | --- | --- | --- | --- | --- | --- | --- | --- | --- | --- |
|  | **Model 1** | | | | **Model 2** | | | | **Model 1** | | | | **Model 2** | | | |
|  | *NW* | *OW* | *OB* | *p-value* | *NW* | *OW* | *OB* | *p-value* | *Lower Q* | *Middle Q* | *Upper Q* | *p-value* | *Lower Q* | *Middle Q* | *Upper Q* | *p-value* |
| n= | 353 | 152 | 60 |  | 353 | 152 | 60 |  | 133 | 253 | 143 |  | 133 | 253 | 143 |  |
| MAP (mmHg) | 74.1 ±0.35 | 77.4 ±0.54 | 82.0 ±0.87 | **<0.001** | 74.2 ±0.37 | 77.3 ±0.55 | 81.5 ±0.96 | **<0.001** | 77.7  ±0.59 | 75.7 ±0.41^U^ | 74.2 ±0.59 | **<0.001** | 75.4 ±0.63 | 75.8 ±0.39 | 76.1 ±0.61 | 0.740 |
| Log Insulin (mg/dl) | 0.72 ±0.01 | 0.88 ±0.01 | 1.04 ±0.02 | **<0.001** | 0.73 ±0.01 | 0.86 ±0.01 | 0.99 ±0.02 | **<0.001** | 0.88 ±0.02^M^ | 0.80 ±0.01 | 0.71 ±0.01 | **<0.001** | 0.77 ±0.01 | 0.81 ±0.01 | 0.80 ±0.01 | 0.356 |
| Waist circum- ference (cm) | 62.6 ±0.27 | 74.5 ±0.42 | 86.0 ±0.67 | **<0.001** | 63.4 ±0.27 | 73.5 ±0.40 | 83.2 ±0.69 | **<0.001** | 76.0 ±0.67 | 67.8 ±0.47 | 61.2 ±0.67 | **<0.001** | 68.6  ±0.29 | 68.3 ±0.18 | 67.6 ±0.28 | 0.054 |
| Log TG/HDL-c (mg/dl) | -0.09 ±0.01 | 0.07 ±0.01 | 0.26 ±0.02 | **<0.001** | -0.08 ±0.01 | 0.06 ±0.01 | 0.23 ±0.03 | **<0.001** | 0.09 ±0.02 | -0.01 ±0.01 | -0.10 ±0.02 | **<0.001** | 0.002 ±0.02 | -0.01 ±0.01 | -0.02 ±0.02 | 0.776 |
| CMRI | -0.87 ±0.06 | 0.99 ±0.09 | 2.56 ±0.15 | **<0.001** | -0.75 ±0.06 | 0.86 ±0.09 | 2.13 ±0.16 | **<0.001** | 1.07 ±0.13 | -0.006 ±0.09 | -1.09 ±0.13 | **<0.001** | -0.03 ±0.10 | 0.06 ±0.06 | -0.12 ±0.09 | 0.213 |
| Data are presented as marginal estimated mean ± SE.  MAP= mean arterial blood pressure (DBP + [0.333 x (SBP - DBP)]); Log Insulin= logarithm of fasting insulin; Log TG/HDL-c = logarithm of triglyceride to high density lipoprotein cholesterol ratio. CMRI = cardiometabolic risk index.  Categories of body mass index (BMI) are Normal Weight (NW), Overweight (OW) and Obesity (OB) according to gender-and-age-specific cut-offs defined by Cole and Lobstein. Categories of dynamometry/weight are Lower Q (representing 1^st^ quartile), Middle Q (2^nd^ and 3^rd^ quartiles), and Upper Q (4th quartile).  Model 1 controlling for age. All the pairwise mean comparisons using Bonferroni post-hoc test were statistically significant (p<0.001), NW<OW<OB for BMI categories and Lower Q>Middle Q>Upper Q for dynamometry/weight, except for superscript letter.  Model 2 further adjustments for dynamometry/weight to BMI and for BMI to dynamometry/weight. All the pairwise mean comparisons using Bonferroni post-hoc test were statistically significant (NW<OW<OB) for BMI categories. | | | | | | | | | | | | | | | | |

|  | **Body Mass Index** | | | | | | | | **Dynamometry/weight** | | | | | | | | |
| --- | --- | --- | --- | --- | --- | --- | --- | --- | --- | --- | --- | --- | --- | --- | --- | --- | --- |
|  | **Model 1** | | | | **Model 2** | | | | **Model 1** | | | | **Model 2** | | | | |
|  | *NW* | *OW* | *OB* | *p-value* | *NW* | *OW* | *OB* | *p-value* | *Lower Q* | *Middle Q* | *Upper Q* | *p-value* | *Lower Q* | *Middle Q* | *Upper Q* | | *p-value* |
| n= | 342 | 147 | 57 |  | 342 | 147 | 57 |  | 93 | 324 | 113 |  | 93 | 324 | | 113 |  |
| MAP (mmHg) | 73.1 ±0.34 | 76.7 ±0.56 | 81.1 ±0.88 | **<0.001** | 72.9 ±0.36 | 77.1 ±0.59 | 82.1 ±0.97 | **<0.001** | 76.2 ±0.59^M^ | 74.7 ±0.41^U^ | 73.6 ±0.59 | **0.007** | 73.5 ±0.63 | 75.0 ±0.39 | | 75.7 ±0.60 | 0.063 |
| Log Insulin (mg/dl) | 0.80 ±0.01 | 0.96 ±0.01 | 1.17 ±0.02 | **<0.001** | 0.80 ±0.01 | 0.95 ±0.01 | 1.16 ±0.03 | **<0.001** | 0.97 ±0.01 | 0.86 ±0.01 | 0.79 ±0.01 | **<0.001** | 0.85 ±0.01 | 0.88 ±0.01 | | 0.89 ±0.01 | 0.270 |
| Waist circum- ference (cm) | 62.2 ±0.27 | 73.6 ±0.44 | 83.7 ±0.70 | **<0.001** | 62.9 ±0.27 | 72.5 ±0.44 | 81.5 ±0.72 | **<0.001** | 74.7 ±0.63 | 66.2 ±0.44 | 60.8 ±0.63 | **<0.001** | 67.4  ±0.32 | 67.1 ±0.20 | | 66.4 ±0.31 | 0.094 |
| Log TG/HDL‑c (mg/dl) | 0.003 ±0.01 | 0.11 ±0.02 | 0.34 ±0.03 | **<0.001** | 0.01 ±0.01 | 0.10 ±0.02 | 0.33 ±0.03 | **<0.001** | 0.17 ±0.02 | 0.04 ±0.01^U^ | 0.03 ±0.02 | **<0.001** | 0.07 ±0.02 | 0.05 ±0.01 | | 0.08 ±0.02 | 0.363 |
| CMRI | -0.79 ±0.06 | 1.03 ±0.10 | 2.83 ±0.16 | **<0.001** | -0.72 ±0.06 | 0.94 ±0.11 | 2.67 ±0.18 | **<0.001** | 1.15 ±0.13 | -0.12 ±0.09 | -0.90 ±0.13 | **<0.001** | -0.11 ±0.10 | 0.01 ±0.06 | | 0.08 ±0.09 | 0.422 |
| Data are presented as marginal estimated mean ± SE.  MAP= mean arterial blood pressure (DBP + [0.333 x (SBP - DBP)]); Log Insulin= logarithm of fasting insulin; Log TG/HDL-c = logarithm of triglyceride to high density lipoprotein cholesterol ratio. CMRI = cardiometabolic risk index.  Categories of body mass index (BMI) are Normal Weight (NW), Overweight (OW) and Obesity (OB) according to gender-and-age-specific cut-offs defined by Cole and Lobstein. Categories of dynamometry/weight are Lower Q (representing 1^st^ quartile), Middle Q (2^nd^ and 3^rd^ quartiles), and Upper Q (4th quartile).  Model 1 controlling for age. All the pairwise mean comparisons using Bonferroni post-hoc test were statistically significant (p<0.001), NW<OW<OB for BMI categories and Lower Q>Middle Q>Upper Q for dynamometry/weight, except for superscript letters.  Model 2 further adjustments for dynamometry/weight to BMI and for BMI to dynamometry/weight. All the pairwise mean comparisons using Bonferroni post-hoc test were statistically significant (NW<OW<OB) for BMI categories. | | | | | | | | | | | | | | | | | |

**Table B. ANCOVA model testing mean differences in cardiometabolic risk factors by body composition and dynamometry/weight categories in girls.**

**Table C. ANCOVA model testing mean differences in cardiometabolic risk factors by body composition and standing long jump categories in boys.**

|  | **Body Mass Index** | | | | | | | | **Standing Long Jump** | | | | | | | |
| --- | --- | --- | --- | --- | --- | --- | --- | --- | --- | --- | --- | --- | --- | --- | --- | --- |
|  | **Model 1** | | | | **Model 2** | | | | **Model 1** | | | | **Model 2** | | | |
|  | *NW* | *OW* | *OB* | *p-value* | *NW* | *OW* | *OB* | *p-value* | *Lower Q* | *Middle Q* | *Upper Q* | *p-value* | *Lower Q* | *Middle Q* | *Upper Q* | *p-value* |
| n= | 353 | 152 | 60 |  | 353 | 152 | 60 |  | 133 | 253 | 143 |  | 133 | 253 | 143 |  |
| MAP (mmHg) | 74.1 ±0.35 | 77.4 ±0.54 | 82.0 ±0.87 | **<0.001** | 74.2 ±0.36 | 77.3 ±0.55 | 81.8 ±0.89 | **<0.001** | 77.2  ±0.60^M^ | 75.8 ±0.42^U^ | 74.6 ±0.61 | **0.011** | 75.9 ±0.58 | 75.7 ±0.39 | 76.2 ±0.60 | 0.785 |
| Log Insulin (mg/dl) | 0.72 ±0.01 | 0.88 ±0.01 | 1.04 ±0.02 | **<0.001** | 0.73 ±0.01 | 0.87 ±0.01 | 1.01 ±0.02 | **<0.001** | 0.89 ±0.02 | 0.79 ±0.01 | 0.71 ±0.01 | **<0.001** | 0.83 ±0.01 | 0.79 ±0.01 | 0.77 ±0.01 | 0.108 |
| Waist circum- ference (cm) | 62.6 ±0.27 | 74.5 ±0.42 | 86.0 ±0.67 | **<0.001** | 62.8 ±0.28 | 74.1 ±0.42 | 85.3 ±0.68 | **<0.001** | 72.3 ±0.74 | 68.7 ±0.51 | 62.4 ±0.75 | **<0.001** | 66.5  ±0.27 | 68.4 ±0.18 | 67.8 ±0.28 | 0.131 |
| Log TG/HDL-c (mg/dl) | -0.09 ±0.01 | 0.07 ±0.01 | 0.26 ±0.02 | **<0.001** | -0.08 ±0.01 | 0.06 ±0.01 | 0.23 ±0.02 | **<0.001** | 0.07 ±0.02 | 0.000 ±0.01 | -0.10 ±0.02 | **<0.001** | 0.01 ±0.02 | -0.003 ±0.01 | -0.04 ±0.02 | 0.156 |
| CMRI | -0.87 ±0.06 | 0.99 ±0.09 | 2.56 ±0.15 | **<0.001** | -0.82 ±0.06 | 0.92 ±0.09 | 2.40 ±0.15 | **<0.001** | 0.86 ±0.13 | 0.03 ±0.09 | -0.93 ±0.13 | **<0.001** | 0.16 ±0.09 | -0.01 ±0.06 | -0.16 ±0.09 | 0.058 |
| Data are presented as marginal estimated mean ± SE.  MAP= mean arterial blood pressure (DBP + [0.333 x (SBP - DBP)]); Log Insulin= logarithm of fasting insulin; Log TG/HDL-c = logarithm of triglyceride to high density lipoprotein cholesterol ratio. CMRI = cardiometabolic risk index.  Categories of body mass index (BMI) are Normal Weight (NW), Overweight (OW) and Obesity (OB) according to gender-and-age-specific cut-offs defined by Cole and Lobstein. Categories of standing long jump are Lower Q (representing 1^st^ quartile), Middle Q (2^nd^ and 3^rd^ quartiles), and Upper Q (4th quartile).  Model 1 controlling for age. All the pairwise mean comparisons using Bonferroni post-hoc test were statistically significant (p<0.001), NW<OW<OB for BMI categories and Lower Q>Middle Q>Upper Q for standing long jump, except for superscript letters.  Model 2 further adjustments for standing long jump to BMI and for BMI to standing long jump. All the pairwise mean comparisons using Bonferroni post-hoc test were statistically significant (NW<OW<OB) for BMI categories. | | | | | | | | | | | | | | | | |

| **Table D. ANCOVA models testing mean differences in cardiometabolic risk factors by body composition and standing long jump categories in girls.**     \|  \| **Body Mass Index** \| \| \| \| \| \| \| \| **Standing Long Jump** \| \| \| \| \| \| \| \| \| \| --- \| --- \| --- \| --- \| --- \| --- \| --- \| --- \| --- \| --- \| --- \| --- \| --- \| --- \| --- \| --- \| --- \| --- \| \|  \| **Model 1** \| \| \| \| **Model 2** \| \| \| \| **Model 1** \| \| \| \| **Model 2** \| \| \| \| \| \|  \| *NW* \| *OW* \| *OB* \| *p-value* \| *NW* \| *OW* \| *OB* \| *p-value* \| *Lower Q* \| *Middle Q* \| *Upper Q* \| *p-value* \| *Lower Q* \| *Middle Q* \| *Upper Q* \| \| *p- value* \| \| n= \| 342 \| 147 \| 57 \|  \| 342 \| 147 \| 57 \|  \| 93 \| 324 \| 113 \|  \| 93 \| 324 \| \| 113 \|  \| \| MAP (mmHg) \| 73.1 ±0.34 \| 76.7 ±0.56 \| 81.1 ±0.88 \| **<0.001** \| 73.1 ±0.34 \| 76.6 ±0.57 \| 81.4 ±0.90 \| **<0.001** \| 76.2 ±0.58^M^ \| 74.6 ±0.42^U^ \| 73.6 ±0.59 \| **0.009** \| 75.1 ±0.56 \| 74.7 ±0.39 \| \| 74.6 ±0.57 \| 0.808 \| \| Log Insulin (mg/dl) \| 0.80 ±0.01 \| 0.96 ±0.01 \| 1.17 ±0.02 \| **<0.001** \| 0.80 ±0.01 \| 0.96 ±0.01 \| 1.17 ±0.02 \| **<0.001** \| 0.92 ±0.01^M^ \| 0.87 ±0.01 \| 0.82 ±0.01 \| **0.001** \| 0.86 ±0.01 \| 0.88 ±0.01 \| \| 0.87 ±0.01 \| 0.849 \| \| Waist circum- ference (cm) \| 62.2 ±0.27 \| 73.6 ±0.44 \| 83.7 ±0.70 \| **<0.001** \| 62.2 ±0.27 \| 73.3 ±0.44 \| 83.5 ±0.70 \| **<0.001** \| 70.9 ±0.72 \| 66.8 ±0.51 \| 63.4 ±0.73 \| **<0.001** \| 67.3  ±0.28 \| 67.0 ±0.20 \| \| 66.6 ±0.29 \| 0.249 \| \| Log TG/HDL-c (mg/dl) \| 0.003 ±0.01 \| 0.11 ±0.02 \| 0.34 ±0.03 \| **<0.001** \| 0.003 ±0.01 \| 0.11 ±0.02 \| 0.35 ±0.03 \| **<0.001** \| 0.12 ±0.02 \| 0.05 ±0.01^U^ \| 0.02 ±0.02 \| **0.002** \| 0.07 ±0.01 \| 0.05 ±0.01 \| \| 0.07 ±0.01 \| 0.445 \| \| CMRI \| -0.79 ±0.06 \| 1.03 ±0.10 \| 2.83 ±0.16 \| **<0.001** \| -0.79 ±0.06 \| 1.01 ±0.10 \| 2.84 ±0.17 \| **<0.001** \| 0.60 ±0.14 \| -0.04 ±0.10 \| -0.55 ±0.14 \| **<0.001** \| 0.02 ±0.08 \| -0.02 ±0.06 \| \| -0.009 ±0.09 \| 0.896 \| \| Data are presented as marginal estimated mean ± SE.  MAP= mean arterial blood pressure (DBP + [0.333 x (SBP - DBP)]); Log Insulin= logarithm of fasting insulin; Log TG/HDL-c = logarithm of triglyceride to high density lipoprotein cholesterol ratio. CMRI = cardiometabolic risk index.  Categories of body mass index (BMI) are Normal Weight (NW), Overweight (OW) and Obesity (OB) according to gender-and-age-specific cut-offs defined by Cole and Lobstein. Categories of standing long jump are Lower Q (representing 1^st^ quartile), Middle Q (2^nd^ and 3^rd^ quartiles), and Upper Q (4th quartile).  Model 1 controlling for age. All the pairwise mean comparisons using Bonferroni post-hoc test were statistically significant (p<0.001), NW<OW<OB for BMI categories and Lower Q>Middle Q>Upper Q for standing long jump, except for superscript letters.  Model 2 further adjustments for standing long jump to BMI and for BMI to standing long jump. All the pairwise mean comparisons using Bonferroni post-hoc test were statistically significant (NW<OW<OB) for BMI categories. \| \| \| \| \| \| \| \| \| \| \| \| \| \| \| \| \| \|     **Table E. ANCOVA model testing mean differences in cardiometabolic risk factors by body mass index and adjusted standing long jump categories.** | | | | | | | | | | | | | | | | |  |  |
| --- | --- | --- | --- | --- | --- | --- | --- | --- | --- | --- | --- | --- | --- | --- | --- | --- | --- | --- | --- | --- | --- | --- | --- | --- | --- | --- | --- | --- | --- | --- | --- | --- | --- | --- | --- | --- | --- | --- | --- | --- | --- | --- | --- | --- | --- | --- | --- | --- | --- | --- | --- | --- | --- | --- | --- | --- | --- | --- | --- | --- | --- | --- | --- | --- | --- | --- | --- | --- | --- | --- | --- | --- | --- | --- | --- | --- | --- | --- | --- | --- | --- | --- | --- | --- | --- | --- | --- | --- | --- | --- | --- | --- | --- | --- | --- | --- | --- | --- | --- | --- | --- | --- | --- | --- | --- | --- | --- | --- | --- | --- | --- | --- | --- | --- | --- | --- | --- | --- | --- | --- | --- | --- | --- | --- | --- | --- | --- | --- | --- | --- | --- | --- | --- | --- | --- | --- | --- | --- | --- | --- | --- | --- | --- | --- | --- | --- | --- | --- | --- | --- | --- | --- | --- | --- | --- | --- | --- | --- | --- | --- | --- | --- | --- | --- | --- | --- | --- | --- | --- | --- | --- | --- | --- | --- | --- | --- | --- | --- | --- | --- | --- | --- | --- | --- | --- | --- | --- | --- | --- | --- | --- | --- | --- | --- | --- | --- | --- | --- |
|  | | **Body Mass Index** | | | | | | | | **SLJ^a^** | | | | | | | | |
|  | | **Model 1** | | | | **Model 2** | | | | **Model 1** | | | | **Model 2** | | | |  |
|  | | *NW* | *OW* | *OB* | *p-value* | *NW* | *OW* | *OB* | *p-value* | *Lower Q* | *Middle Q* | *Upper Q* | *p-value* | *Lower Q* | *Middle Q* | *Upper Q* | *p-value* | |
| n= | | 744 | 296 | 118 |  | 744 | 296 | 118 |  | 285 | 575 | 286 |  | 285 | 575 | 286 |  | |
| MAP (mmHg) | | 73.6  ±0.24 | 77.0  ±0.38 | 81.5  ±0.62 | **<0.001** | 73.6  ±0.25 | 77.0  ±0.40 | 81.6  ±0.64 | **<0.001** | 76.7  ±0.43 | 75.3  ±0.29 | 74.0  ±0.43 | **<0.001** | 75.5  ±0.41^MU^ | 75.2  ±0.28^U^ | 75.3  ±0.42 | 0.836 | |
| Log Insulin (mg/dl) | | 0.76  ±0.01 | 0.92  ±0.01 | 1.10  ±0.02 | **<0.001** | 0.76  ±0.01 | 0.92  ±0.01 | 1.09  ±0.02 | **<0.001** | 0.89  ±0.01 | 0.85  ±0.01 | 0.76  ±0.01 | **<0.001** | 0.84  ±0.01^MU^ | 0.84  ±0.01^U^ | 0.83  ±0.01 | 0.671 | |
| Waist circum- ference (cm) | | 62.4  ±0.19 | 74.0  ±0.30 | 84.9  ±0.48 | **<0.001** | 62.5  ±0.19 | 73.7  ±0.31 | 84.4  ±0.49 | **<0.001** | 71.7  ±0.53 | 68.0  ±0.37 | 62.8  ±0.54 | **<0.001** | 67.8  ±0.20^MU^ | 67.8  ±0.14^U^ | 67.2  ±0.21 | **0.039** | |
| Log TG/HDL –c (mg/dl) | | -0.04  ±0.01 | 0.09  ±0.01 | 0.30  ±0.02 | **<0.001** | -0.04  ±0.01 | 0.09  ±0.01 | 0.29  ±0.02 | **<0.001** | 0.09  ±0.01 | 0.03  ±0.01 | -0.04  ±0.01 | **<0.001** | 0.04  ±0.01^MU^ | 0.03  ±0.01^U^ | 0.02  ±0.01 | 0.591 | |
| CMRI | | -0.83  ±0.04 | 1.01  ±0.07 | 2.68  ±0.11 | **<0.001** | -0.81  ±0.05 | 0.96  ±0.07 | 2.61  ±0.12 | **<0.001** | 0.66  ±0.10 | 0.05  ±0.07 | -0.80  ±0.10 | **<0.001** | 0.05  ±0.07^MU^ | 0.01  ±0.04 | -0.09  ±0.07 | 0.366 | |
| Data are presented as marginal estimated mean ± SE.  MAP= mean arterial blood pressure (DBP + {0.333 x (SBP - DBP)}); Log Insulin= logarithm of fasting insulin; Log TG/HDL-c = logarithm of triglyceride to high density lipoprotein cholesterol ratio. CMRI = cardiometabolic risk index.  ^a^SLJ was adjusted by allometric parameters defined by Jaric (SLJ/weight^0^)  Categories of body mass index (BMI) are Normal Weight (NW), Overweight (OW) and Obesity (OB) according to gender-and-age-specific cut-offs defined by Cole and Lobstein. Categories of SLJ are Lower Q (representing 1st quartile), Middle Q (2nd and 3rd quartiles), and Upper Q (4th quartile).  Model 1 controlling for age and sex. All the pairwise mean comparisons using Bonferroni post-hoc test were statistically significant (p<0.001) (NW<OW<OB for BMI categories; and Lower Q>Middle Q>Upper Q for SLJ).  Model 2 further adjustments for SLJ to BMI and for BMI to SLJ. All the pairwise mean comparison using Bonferroni post-hoc test were statistically significant (NW<OW<OB for BMI categories), except for superscript letters in standing long jump categories. | | | | | | | | | | | | | | | | | |  |

**Table F. ANCOVA model testing mean differences in cardiometabolic risk factors by body mass index and adjusted dynamometry categories.**

|  | **Body Mass Index** | | | | | | | | **Dynamometry^a^** | | | | | | | | |  |
| --- | --- | --- | --- | --- | --- | --- | --- | --- | --- | --- | --- | --- | --- | --- | --- | --- | --- | --- |
|  | **Model 1** | | | | **Model 2** | | | | **Model 1** | | | | **Model 2** | | | |  |  |
|  | *N* | *OW* | *OB* | *p-value* | *N* | *OW* | *OB* | *p-value* | *Lower Q* | *Middle Q* | *Upper Q* | *p-value* | *Lower Q* | *Middle Q* | *Upper Q* | *p-value* | | |
| n= | 744 | 296 | 118 |  | 744 | 296 | 118 |  | 285 | 574 | 285 |  | 285 | 574 | 285 |  | | |
| MAP (mmHg) | 73.6  ±0.24 | 77.0  ±0.38 | 81.5  ±0.62 | **<0.001** | 73.6 ±0.25 | 77.2  ±0.39 | 81.8  ±0.64 | **<0.001** | 75.2± 0.42^MU^ | 75.6  ±0.30^U^ | 74.7  ±0.43 | 0.278 | 74.1± 0.41 | 75.6  ±0.28^U^ | 76.0  ±0.41 | **0.002** | | |
| Log Insulin (mg/dl) | 0.76  ±0.01 | 0.92  ±0.01 | 1.10  ±0.02 | **<0.001** | 0.77 ±0.01 | 0.92 ±0.01 | 1.10  ±0.02 | **<0.001** | 0.87  ±0.01 | 0.83  ±0.01^U^ | 0.81  ±0.01 | **0.004** | 0.82± 0.01^M^ | 0.83  ±0.01^U^ | 0.87  ±0.01 | **0.029** | | |
| Waist circum- ference (cm) | 62.4  ±0.19 | 74.0  ±0.30 | 84.9  ±0.48 | **<0.001** | 62.5 ±0.19 | 73.8 ±0.31 | 84.4  ±0.49 | **<0.001** | 71.4  ±0.54 | 67.6  ±0.37 | 63.7  ±0.55 | **<0.001** | 67.8± 0.20^MU^ | 67.6  ±0.14^U^ | 67.5  ±0.21 | 0.479 | | |
| Log TG/HDL –c (mg/dl) | -0.04  ±0.01 | 0.09  ±0.01 | 0.30  ±0.02 | **<0.001** | -0.04 ±0.01 | 0.09  ±0.01 | 0.30  ±0.02 | **<0.001** | 0.09  ±0.02 | 0.03  ±0.01 | -0.04  ±0.02 | **<0.001** | 0.04± 0.01^MU^ | 0.03  ±0.01^U^ | 0.01  ±0.01 | 0.524 | | |
| CMRI | -0.83  ±0.04 | 1.01  ±0.07 | 2.68  ±0.11 | **<0.001** | -0.82 ±0.05 | 1.00  ±0.07 | 2.67  ±0.12 | **<0.001** | 0.47  ±0.10 | 0.003  ±0.07 | -0.57  ±0.10 | **<0.001** | -0.10± 0.06^MU^ | -0.001  ±0.04^U^ | 0.09  ±0.07 | 0.132 | | |

Data are presented as marginal estimated mean ± SE.

MAP= mean arterial blood pressure (DBP + {0.333 x (SBP - DBP)}); Log Insulin= logarithm of fasting insulin; Log TG/HDL-c = logarithm of triglyceride to high density lipoprotein cholesterol ratio. CMRI = cardiometabolic risk index.

^a^ Dynamometry was adjusted by allometric parameters defined by Jaric (dynamometry/weight^0.67^)

Categories of body mass index (BMI) are Normal Weight (NW), Overweight (OW) and Obesity (OB) according to gender-and-age-specific cut-offs defined by Cole and Lobstein. Categories of dynamometry are Lower Q (representing 1st quartile), Middle Q (2nd and 3rd quartiles), and Upper Q (4th quartile).

Model 1 controlling for age and sex. All the pairwise mean comparisons using Bonferroni post-hoc test were statistically significant (p<0.001) (NW<OW<OB for BMI categories; and Lower Q>Middle Q>Upper Q for dynamometry), except for superscript letters.

Model 2 further adjustments for dynamometry to BMI and for BMI to dynamometry. All the pairwise mean comparison using Bonferroni post-hoc test were statistically significant (NW<OW<OB for BMI categories), except for superscript letters in dynamometry categories.

**Table G. ANCOVA model testing mean differences in cardiometabolic risk factors by body mass index and adjusted muscular fitness categories.**

|  | **Body Mass Index** | | | | | | | | **Muscular fitness^a^** | | | | | | | | |
| --- | --- | --- | --- | --- | --- | --- | --- | --- | --- | --- | --- | --- | --- | --- | --- | --- | --- |
|  | **Model 1** | | | | **Model 2** | | | | **Model 1** | | | | **Model 2** | | | |  |
|  | *NW* | *OW* | *OB* | *p-value* | *NW* | *OW* | *OB* | *p-value* | *Lower Q* | *Middle Q* | *Upper Q* | *p-value* | *Lower Q* | *Middle Q* | *Upper Q* | *p-value* | |
| n= | 744 | 296 | 118 |  | 744 | 296 | 118 |  | 279 | 558 | 275 |  | 285 | 574 | 285 |  | |
| MAP (mmHg) | 73.6  ±0.24 | 77.0  ±0.38 | 81.5  ±0.62 | **<0.001** | 73.6  ±0.25 | 77.1  ±0.40 | 81.7  ±0.65 | **<0.001** | 76.4  ±0.43^M^ | 75.3  ±0.30 ^M^ | 74.2  ±0.43 | **0.001** | 75.0  ±0.42^MU^ | 75.3  ±0.28^U^ | 75.6  ±0.42 | 0.597 | |
| Log Insulin (mg/dl) | 0.76  ±0.01 | 0.92  ±0.01 | 1.10  ±0.02 | **<0.001** | 0.77 ±0.01 | 0.92 ±0.01 | 1.09  ±0.02 | **<0.001** | 0.91  ±0.01 | 0.83  ±0.01 | 0.78  ±0.01 | **<0.001** | 0.84  ±0.01^MU^ | 0.83  ±0.01^U^ | 0.85  ±0.01 | 0.319 | |
| Waist circum- ference (cm) | 62.4  ±0.19 | 74.0  ±0.30 | 84.9  ±0.48 | **<0.001** | 62.6 ±0.20 | 73.6 ±0.31 | 84.1  ±0.50 | **<0.001** | 72.6  ±0.52 | 67.6  ±0.36 | 62.5  ±0.53 | **<0.001** | 67.9  ±0.21^MU^ | 67.6  ±0.14^U^ | 67.3  ±0.21 | 0.145 | |
| Log TG/HDL –c (mg/dl) | -0.04  ±0.01 | 0.09  ±0.01 | 0.30  ±0.02 | **<0.001** | -0.04 ±0.01 | 0.09  ±0.01 | 0.29  ±0.02 | **<0.001** | 0.09  ±0.02 | 0.04  ±0.01 | -0.07  ±0.02 | **<0.001** | 0.03  ±0.01^MU^ | 0.04  ±0.01 | -0.003  ±0.01 | **0.038** | |
| CMRI | -0.83  ±0.04 | 1.01  ±0.07 | 2.68  ±0.11 | **<0.001** | -0.80 ±0.05 | 0.97  ±0.07 | 2.61  ±0.12 | **<0.001** | 0.74  ±0.10 | 0.01  ±0.07 | -0.79  ±0.10 | **<0.001** | 0.01  ±0.07^MU^ | 0.001  ±0.04^U^ | -0.03  ±0.07 | 0.893 | |

Data are presented as marginal estimated mean ± SE.

MAP= mean arterial blood pressure (DBP + {0.333 x (SBP - DBP)}); Log Insulin= logarithm of fasting insulin; Log TG/HDL-c = logarithm of triglyceride to high density lipoprotein cholesterol ratio. CMRI = cardiometabolic risk index.

^a^ Muscular fitness= sum of standardized z score of dynamometry/weight^0.67^ and SLJ/weight^0^ , according to allometric parameters defined by Jaric.

Categories of body mass index (BMI) are Normal Weight (NW), Overweight (OW) and Obesity (OB) according to gender-and-age-specific cut-offs defined by Cole and Lobstein. Categories of muscular fitness are Lower Q (representing 1st quartile), Middle Q (2nd and 3rd quartiles), and Upper Q (4th quartile).

Model 1 controlling for age and sex. All the pairwise mean comparisons using Bonferroni post-hoc test were statistically significant (p<0.001) (NW<OW<OB for BMI categories, except for superscript letters in muscular fitness categories).

Model 2 further adjustments for muscular fitness to BMI and for BMI to muscular fitness. All the pairwise mean comparison using Bonferroni post-hoc test were statistically significant (NW<OW<OB for BMI categories), except for superscript letters in muscular fitness categories.
