# Supplementary figures and images for: BMI as a Mediator of the Relationship between Muscular Fitness and Cardiometabolic Risk in Children: A Mediation Analysis
Source: PLoS One. 2015 Jan 15;10(1):e0116506. doi: 10.1371/journal.pone.0116506 (PMC4295865; doi:10.1371/journal.pone.0116506)

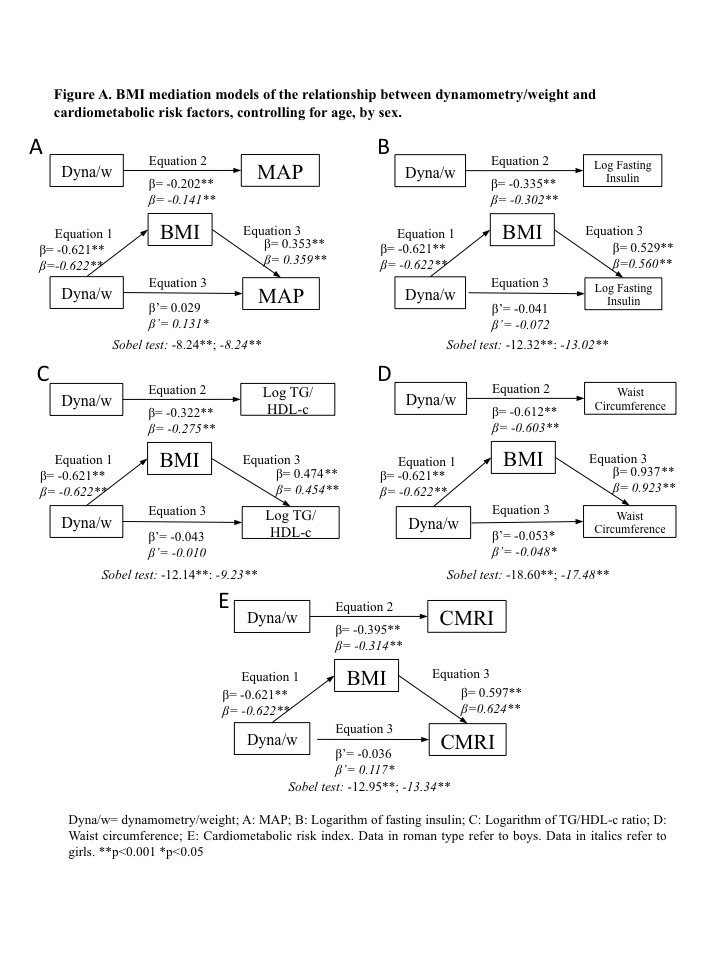

Supplement: S1 File — B Fig. BMI mediation models of the relationship between standing long jump and cardiometabolic risk factors, controlling for age, by sex. A Table. ANCOVA model testing mean differences in cardiometabolic risk factors by body composition and dynamometry/weight categories in boys. B Table. ANCOVA model testing mean differences in cardiometabolic risk factors by body composition and dynamometry/weight categories in girls. C Table. ANCOVA model testing mean differences in cardiometabolic risk factors by body composition and standing long jump categories in boys. D Table. ANCOVA models testing mean differences in cardiometabolic risk factors by body composition and standing long jump categories in girls. E Table. ANCOVA model testing mean differences in cardiometabolic risk factors by body mass index and adjusted standing long jump categories. SLJ was adjusted by allometric parameters defined by Jaric (SLJ/weight0). F Table. ANCOVA model testing mean differences in cardiometabolic risk factors by body mass index and adjusted dynamometry categories. Dynamometry was adjusted by allometric parameters defined by Jaric (dynamometry/weight0.67). G Table. ANCOVA model testing mean differences in cardiometabolic risk factors by body mass index and adjusted muscular fitness categories. Muscular fitness = sum of standardized z score of dynamometry/weight0.67 and SLJ/weight0, according to allometric parameters defined by Jaric. (ZIP) [file pone.0116506.s001.zip › S1 File/Figure A.tiff]

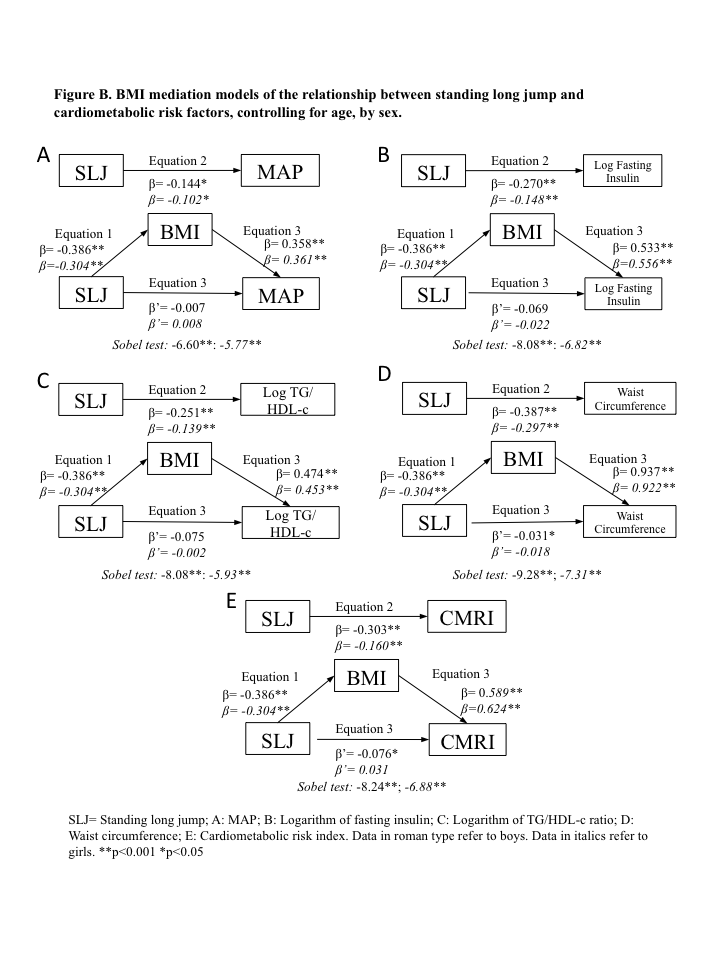

Supplement: S1 File — B Fig. BMI mediation models of the relationship between standing long jump and cardiometabolic risk factors, controlling for age, by sex. A Table. ANCOVA model testing mean differences in cardiometabolic risk factors by body composition and dynamometry/weight categories in boys. B Table. ANCOVA model testing mean differences in cardiometabolic risk factors by body composition and dynamometry/weight categories in girls. C Table. ANCOVA model testing mean differences in cardiometabolic risk factors by body composition and standing long jump categories in boys. D Table. ANCOVA models testing mean differences in cardiometabolic risk factors by body composition and standing long jump categories in girls. E Table. ANCOVA model testing mean differences in cardiometabolic risk factors by body mass index and adjusted standing long jump categories. SLJ was adjusted by allometric parameters defined by Jaric (SLJ/weight0). F Table. ANCOVA model testing mean differences in cardiometabolic risk factors by body mass index and adjusted dynamometry categories. Dynamometry was adjusted by allometric parameters defined by Jaric (dynamometry/weight0.67). G Table. ANCOVA model testing mean differences in cardiometabolic risk factors by body mass index and adjusted muscular fitness categories. Muscular fitness = sum of standardized z score of dynamometry/weight0.67 and SLJ/weight0, according to allometric parameters defined by Jaric. (ZIP) [file pone.0116506.s001.zip › S1 File/Figure B.tiff]
